# Clinical Implications of Cell-Free DNA in Managing *BRAF* V600E Mutation-Positive Colorectal Cancer

**DOI:** 10.3390/genes16030275

**Published:** 2025-02-25

**Authors:** Takuma Iwai, Takeshi Yamada, Kay Uehara, Seiichi Shinji, Akihisa Matsuda, Yasuyuki Yokoyama, Goro Takahashi, Toshimitsu Miyasaka, Hiroshi Yoshida

**Affiliations:** Department of Gastroenterological Surgery, Nippon Medical School, 1-1-5 Sendagi, Bunkyo-ku, Tokyo 113-8602, Japan; y-tak@nms.ac.jp (T.Y.); kay-uehara@nms.ac.jp (K.U.); s-shinji@nms.ac.jp (S.S.); a-matsu@nms.ac.jp (A.M.); y-yokoyama@nms.ac.jp (Y.Y.); s9057@nms.ac.jp (G.T.); miyasaka@nms.ac.jp (T.M.); hiroshiy@nms.ac.jp (H.Y.)

**Keywords:** cell-free DNA, liquid biopsy, *BRAF* V600E, colorectal cancer

## Abstract

**Background/Objectives:** *BRAF*^V600E^-mutant colorectal cancer (CRC) is associated with poor prognosis, and despite the introduction of BEACON therapy, significant treatment challenges remain. This study investigates the clinical utility of *BRAF*^V600E^ in cell-free DNA (cfDNA *BRAF*^V600E^) as a biomarker for real-time treatment monitoring in metastatic cases and for evaluating minimal residual disease (MRD) after curative resection. **Methods**: This single-center, prospective observational study included 37 patients with *BRAF*^V600E^-mutant CRC treated at Nippon Medical School Hospital between April 2017 and June 2024. Patients were divided into two cohorts: Cohort 1 (Stage IV cases): Evaluated cfDNA *BRAF*^V600E^ for treatment monitoring. Cohort 2 (Stage I–III curatively resected cases): Assessed cfDNA *BRAF*^V600E^ for recurrence risk prediction. Blood samples were collected before and during treatment and analyzed using droplet digital PCR (ddPCR) to measure cfDNA *BRAF*^V600E^ levels. **Results**: Cohort 1 (Stage IV, *n* = 14): Pre-treatment cfDNA *BRAF*^V600E^ was detected in 93% of cases. Patients with a decrease in cfDNA *BRAF*^V600E^ variant allele frequency (VAF) after chemotherapy had significantly longer overall survival (511 vs. 189 days, *p* = 0.03) than those without a decrease. Cohort 2 (curatively resected, *n* = 23): cfDNA *BRAF*^V600E^ was detected in 4/23 patients (17.4%) at 1 month post-surgery. cfDNA *BRAF*^V600E^ showed better recurrence prediction compared to CEA (100% vs. 18.8%, *p* = 0.004). Among the seven patients who experienced recurrence, those with postoperative cfDNA *BRAF*^V600E^ positivity had significantly shorter disease-free survival compared to cfDNA *BRAF*^V600E^-negative patients (179 vs. 840 days, *p* = 0.04). **Conclusions**: These findings support cfDNA *BRAF*^V600E^ as a promising biomarker for monitoring treatment response and MRD detection in *BRAF*^V600E^-mutant CRC, reinforcing its role in guiding personalized treatment strategies and postoperative surveillance.

## 1. Introduction

Advances in drug therapy and surgical techniques have extended the median overall survival (OS) of patients with advanced and recurrent colorectal cancer beyond 30 months. However, colorectal cancer with the *BRAF*^V600E^ mutant accounts for 5–10% of all colorectal cancers and has shown poor response to conventional chemotherapy. The median OS of patients with *BRAF*^V600E^-mutant CRC is reported to be approximately 10 months, compared to about 30 months in *BRAF*^V600E^ wild-type cases [1,2], indicating a significantly worse prognosis. In particular, some cases exhibit rapid disease progression, and the transition to second-line treatment is often difficult. The combination therapy of a *BRAF* inhibitor and an anti-*EGFR* antibody (encorafenib + binimetinib + cetuximab or encorafenib + cetuximab, hereafter referred to as the BEACON regimen) has demonstrated efficacy, establishing a new therapeutic option for previously treated patients with unresectable advanced and recurrent *BRAF*^V600E^-mutant CRC [3,4]. However, despite the introduction of the BEACON regimen, the poor prognosis of *BRAF*^V600E^-mutant CRC remains unchanged, necessitating the development of more effective multidisciplinary treatments and therapeutic strategies.

Two major clinical challenges remain in the treatment of *BRAF*^V600E^-mutant CRC.

### 1.1. Real-Time Evaluation of Treatment Response

The monitoring of treatment efficacy in *BRAF*^V600E^-mutant CRC has traditionally relied on tumor markers (CEA, CA19-9) and imaging assessments. However, these indicators have limitations in sensitivity and specificity, making real-time disease monitoring difficult. In particular, evaluating resistance to the BEACON regimen and determining the eligibility for conversion surgery (curative resection after chemotherapy) require a more sensitive and objective biomarker that can accurately and promptly assess treatment efficacy and guide therapeutic decision-making.

### 1.2. Minimal Residual Disease (MRD) Evaluation After Curative Resection

Several studies have reported that the prognosis of *BRAF*^V600E^-mutant CRC remains worse than that of *BRAF*^V600E^ wild-type CRC even after curative resection [5,6]. Furthermore, subgroup analyses of the MOSAIC trial have suggested that the additional benefit of oxaliplatin-based adjuvant therapy is limited in patients with *BRAF*^V600E^ mutations. Pathological evaluation alone has limitations in detecting MRD (minimal residual disease). Therefore, high-sensitivity molecular tools are required to complement pathological assessments. Given the high recurrence risk in *BRAF*^V600E^-mutant CRC, optimizing adjuvant therapy strategies and follow-up protocols based on MRD evaluation is crucial.

Liquid biopsy is a non-invasive technique enabling repeated sampling and analysis of tumor-derived molecular information from body fluids such as blood [7,8,9,10,11]. We have previously reported the utility of cell-free DNA (cfDNA) in monitoring the treatment response of colorectal cancer [12]. cfDNA has a shorter half-life (several hours) than tumor markers (CEA, CA19-9), allowing for more real-time assessment of tumor dynamics [13]. Therefore, cfDNA *BRAF*^V600E^ is expected to serve as a promising biomarker for treatment monitoring and MRD assessment in *BRAF*^V600E^-mutant CRC.

This study aims to evaluate the clinical utility of cfDNA *BRAF*^V600E^ for treatment monitoring in Stage IV patients and MRD assessment in patients after curative resection of *BRAF*^V600E^-mutant CRC.

## 2. Materials and Methods

### 2.1. Study Population and Design

This study was a single-center prospective observational study of patients with *BRAF^V600E^*-mutant colorectal cancer. Patients who underwent surgery or chemotherapy at Nippon Medical School Hospital between April 2017 and June 2024 were included. Patients were enrolled if *BRAF*^V600E^ mutation was detected in surgical specimens or endoscopic biopsy samples. Patients with high-frequency microsatellite instability (MSI-H) were excluded. The study population was classified into two cohorts according to the study objectives:

- Cohort 1 (Stage IV cases): Prediction of treatment response using cfDNA;
- Cohort 2 (Stage I–III curatively resected cases): Minimal residual disease (MRD) assessment using cfDNA

Patients with synchronous malignancies in other organs were excluded to ensure cfDNA analysis reflected the primary tumor and maintained data consistency. The primary tumor location was classified as right-sided (cecum to transverse colon) or left-sided (splenic flexure to the rectum).

### 2.2. Blood Sample Collection and cfDNA Extraction

For Stage IV patients, blood samples were collected simultaneously with routine hematological and biochemical tests for treatment evaluation and subsequently at approximately 4-week intervals, in line with tumor marker (CEA, CA19-9) assessments during follow-up. For curatively resected cases, blood was collected preoperatively and at one month postoperatively. Blood was collected using BD Vacutainer EDTA tubes (Becton Dickinson and Company, Franklin Lakes, NJ, USA) and processed within 3 h by centrifugation to obtain plasma. Plasma samples were stored at −80 °C until cfDNA extraction.

cfDNA was extracted from 1 mL of plasma using Maxwell^®^ RSC cfDNA Plasma Kits (Promega, Madison, WI, USA). The cfDNA concentration was measured using the Qubit quantification assay (Thermo Fisher Scientific, Waltham, MA, USA).

### 2.3. Droplet Digital PCR (ddPCR)

cfDNA samples were adjusted to 1000 ng/mL and analyzed using QX200 Droplet Digital PCR (Bio-Rad Laboratories, Hercules, CA, USA). The presence of the *BRAF*^V600E^ mutation was determined using variant allele frequency (VAF) with a detection threshold of ≥0.1%. In this study, cfDNA *BRAF*^V600E^ refers to the VAF of the *BRAF*^V600E^ mutation detected in plasma cfDNA, calculated as the proportion of mutant alleles relative to total *BRAF*^V600E^ alleles.

### 2.4. Statistical Analysis

Comparisons between groups were performed using Fisher’s exact test or the Mann–Whitney U test. Survival analysis was conducted using the Kaplan–Meier method, and group comparisons were performed using the log-rank test. A *p*-value < 0.05 was considered statistically significant. Statistical analyses were conducted using SPSS software version 27.0 (IBM Japan Ltd., Tokyo, Japan).

### 2.5. Ethical Considerations

This study was conducted in accordance with the Declaration of Helsinki and was approved by the Ethics Committee of Nippon Medical School Hospital (Approval No. 28-01-694). Written informed consent was obtained from all patients.

## 3. Results

Among the 60 patients with *BRAF*^V600E^-mutant CRC identified during the study period, 23 MSI-H cases were excluded, leaving 37 eligible patients. Of these, 14 were Stage IV (Cohort 1), and 23 were Stage I–III curatively resected cases (Cohort 2). The baseline characteristics of each cohort are shown in Table 1 and Table 2.

### 3.1. Cohort 1: Stage IV Cases (n = 14)

#### 3.1.1. First-Line Treatment Response and cfDNA

Among the 14 Stage IV patients, 13 received chemotherapy, while 1 patient was not eligible for aggressive treatment due to poor performance status. The first-line chemotherapy regimens were as follows: FOLFOXIRI + Bevacizumab (*n* = 6), mFOLFOX6 + Bevacizumab (*n* = 5), and FOLFOX6 (*n* = 3).

Pre-treatment cfDNA *BRAF*^V600E^ was detected in 13/14 (93%) of Stage IV cases.

#### 3.1.2. Overall Survival (OS) Based on cfDNA *BRAF*^V600E^ Status

After chemotherapy initiation, patients were divided into two groups based on cfDNA *BRAF*^V600E^ VAF reduction: VAF reduction group (*n* = 5) and non-reduction group (*n* = 9).

**Figure 1:**
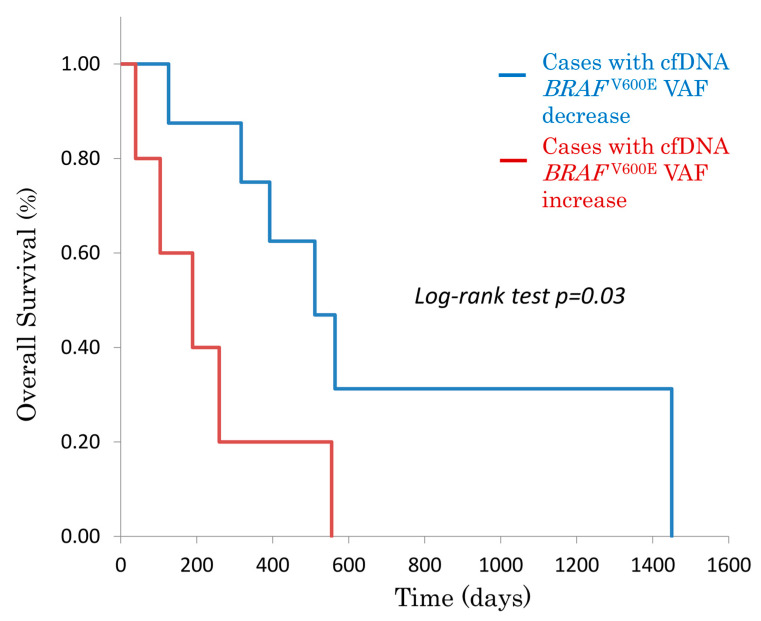
Kaplan–Meier overall survival curve in Stage IV *BRAF* ^V600E^-mutant CRC patients, stratified by cfDNA *BRAF* ^V600E^ variant allele frequency (VAF) changes after chemotherapy. Patients with VAF reduction had significantly longer OS than those without.

The median OS was significantly longer in the VAF reduction group compared to the non-reduction group (median OS: 511 days vs. 189 days, log-rank test, *p* = 0.03) (Figure 1).

#### 3.1.3. Comparison of cfDNA and Tumor Markers in Clinical Practice

##### Case 1: Conversion Surgery After Initial Treatment

**Figure 2:**
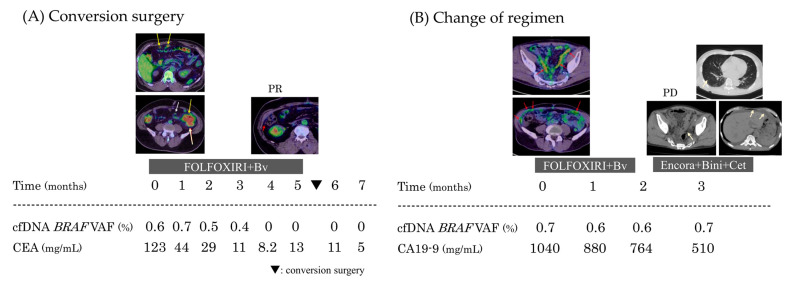
cfDNA and conventional tumor marker dynamics in Stage IV cases. (**A**) The primary tumor and extensive peritoneal dissemination (yellow arrow) responded to treatment, with dissemination becoming localized to the right side (red arrow). At the time when conversion surgery was considered, CEA levels increased, whereas cfDNA *BRAF*^V600E^ remained negative. (**B**) Peritoneal dissemination was observed throughout the abdomen (red arrow). Neither first-line nor second-line therapy was effective, and the patient developed lung metastases and carcinomatous peritonitis (yellow arrow). While cfDNA *BRAF*^V600E^ levels remained unchanged, CA19-9 showed a gradual decreasing trend.

A patient with lower colon cancer and multiple peritoneal metastases showed a partial response (PR) to first-line chemotherapy, and underwent conversion surgery. At the time of partial response (PR) on imaging, the CEA level showed an unexpected rise, whereas cfDNA *BRAF*^V600E^ remained negative. The tumor was surgically removed, and the patient has remained disease-free for more than 10 months after surgery (Figure 2A).

##### Case 2: Timing of Regimen Change

A patient with multiple peritoneal dissemination showed little response to treatment initially and the disease progressed rapidly. While the CA19-9 level decreased over time with primary chemotherapy, there was almost no change in cfDNA *BRAF*^V600E^.

On imaging evaluation, peritoneal dissemination progression and the appearance of lung metastasis were observed, and the patient was transferred to the BEACON regimen, but died of disease about 40 days later (Figure 2B).

### 3.2. Cohort 2: Stage II–III Curatively Resected Cases (n = 23)

Among the 23 patients who underwent curative resection, 9 received adjuvant chemotherapy (regimens: CAPOX:5, FOLFOX:1, UFT/UZEL:3). A total of seven patients (30.4%) developed recurrence, including one with Stage IIa, five with Stage IIIb, and one with Stage IIIc disease. The most common recurrence sites were peritoneal dissemination (*n* = 4), liver (*n* = 2), lung (*n* = 2), and lymph nodes (*n* = 2), with some cases showing multiple recurrence sites.

#### 3.2.1. Postoperative cfDNA BRAF^V600E^ and Recurrence-Free Survival (RFS)

Preoperatively, cfDNA *BRAF*^V600E^ was positive in 1 of 23 cases (4.3%). At 1 month postoperatively, cfDNA *BRAF*^V600E^ was positive in four cases (17.4%). Among the seven recurrence cases, four had postoperative cfDNA *BRAF*^V600E^ positivity.

#### 3.2.2. Comparison with Conventional Tumor Markers

Table 3 summarizes the recurrence prediction performance of cfDNA *BRAF*^V600E^ compared to CEA. cfDNA *BRAF*^V600E^ positivity at 1 month postoperatively was associated with a significantly higher recurrence rate (100% vs. 18.8%, *p* = 0.004). The sensitivity and specificity for recurrence prediction were 57.1% and 100% for cfDNA *BRAF*^V600E^, compared to 28.6% and 81.3% for CEA, respectively. The positive predictive value (PPV) was 100% for cfDNA *BRAF*^V600E^, whereas it was 40.0% for CEA. The negative predictive value (NPV) was 84.2% for cfDNA *BRAF*^V600E^ and 72.2% for CEA.

#### 3.2.3. Postoperative cfDNA *BRAF*^V600E^ Status and DFS in Recurrent Seven Cases

**Figure 3:**
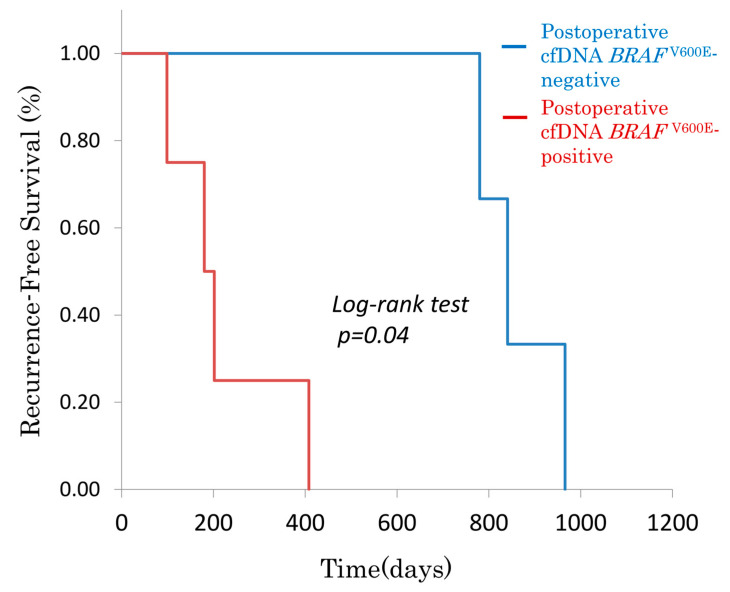
Kaplan-Meier recurrence-free survival curve in curatively resected *BRAF* ^V600E^-mutant CRC patients, stratified by postoperative cfDNA *BRAF*^V600E^ status in seven relapsed cases. Of the seven cases that relapsed after curative resection, the four cases that tested positive for cfDNA *BRAF*^V600E^ 1 month after surgery had a significantly shorter time to relapse than the three cases that tested negative for cfDNA *BRAF*^V600E^.

Among the seven patients who developed recurrence, postoperative cfDNA *BRAF*^V600E^ status was evaluated for its association with DFS (Figure 3). The median DFS was significantly shorter in the cfDNA *BRAF*^V600E^-positive group (*n* = 4; 179 days) compared to the cfDNA *BRAF*^V600E^-negative group (*n* = 3; 840 days) (*p* = 0.04, log-rank test). All four cases with cfDNA *BRAF*^V600E^ positivity experienced recurrence within 6 months after surgery, whereas recurrence occurred beyond 12 months in the cfDNA *BRAF*^V600E^-negative group.

## 4. Discussion

This study demonstrated that cfDNA *BRAF*^V600E^ may serve as a useful biomarker for treatment monitoring and MRD assessment in *BRAF*^V600E^-mutant CRC. In unresectable cases, cfDNA *BRAF*^V600E^ was effective in the real-time evaluation of treatment response, while in curatively resected cases, it was useful for assessing recurrence risk. Previous studies have investigated cfDNA *BRAF*^V600E^ as a biomarker in colorectal cancer [14] and its role in predicting resistance to anti-EGFR therapy [15]. However, this study further builds on these findings by demonstrating the potential utility of cfDNA *BRAF*^V600E^ in guiding treatment decisions and postoperative surveillance, particularly in relation to MRD assessment and conversion surgery selection.

For unresectable *BRAF*^V600E^-mutant CRC, the current standard treatment involves Triplet + Bv or Doublet + Bv as first-line therapy, followed by the BEACON regimen in the second-line setting. The ANCHOR CRC trial evaluated the BEACON regimen as a first-line therapy, reporting an objective response rate (ORR) of 47.8% (95% CI: 37.3–58.5%), thereby meeting its predefined efficacy criteria [16]. Building on this, the BREAKWATER trial, a phase III study comparing encorafenib + cetuximab ± chemotherapy versus chemotherapy alone in the first-line setting, demonstrated a higher ORR in the experimental arm (61%) compared to the control group (40%), further supporting the efficacy of targeted combination therapy [17]. Additionally, for resectable *BRAF*^V600E^-mutant metastatic colorectal cancer, the NEXUS trial, a phase II multicenter study, is currently evaluating the efficacy and safety of perioperative encorafenib + binimetinib + cetuximab. The findings from this trial are expected to provide valuable insights into the role of targeted therapy in the perioperative setting, potentially refining treatment strategies for this aggressive disease subtype [18]. As the treatment landscape for *BRAF*^V600E^-mutant CRC continues to evolve, integrating molecularly targeted approaches with personalized strategies will be crucial in improving patient outcomes and redefining the standard of care.

*BRAF*^V600E^-mutant CRC is aggressive, and conventional tumor markers (CEA, CA19-9) or imaging often fail to accurately assess treatment efficacy [19]. Therefore, a more sensitive and reproducible biomarker is needed. cfDNA *BRAF*^V600E^ can be obtained noninvasively and provides real-time tumor dynamics, making it well suited for treatment response assessment and resistance monitoring. In particular, preoperative evaluation of resectability is crucial in cases where conversion surgery is considered after a favorable response to chemotherapy or in locally advanced disease. A decrease in the variant allele frequency (VAF) of cfDNA B*RAF*^V600E^ mutations has been associated with a better prognosis after resection, suggesting that cfDNA could be an objective indicator for conversion surgery. To further validate the clinical utility of liquid biopsy, the BEETS trial (JACRRO CC-18) has completed data collection, investigating the role of liquid biopsy in metastatic *BRAF*^V600E^-mutant CRC treated with the BEACON regimen [20]. Moving forward, the findings from this study are expected to establish liquid biopsy as a valuable tool for treatment monitoring and personalized therapy in clinical practice.

With the recent inclusion of *RAS* and *BRAF*^V600E^ mutation testing under health insurance coverage, an increasing number of cases are being diagnosed as *BRAF*^V600E^ mutation-positive based on the analysis of resected specimens. In this study, 7 out of 23 patients who underwent curative resection experienced recurrence, and among those who were cfDNA *BRAF*^V600E^-positive postoperatively, disease progression after recurrence was notably rapid. This suggests that postoperative minimal residual disease (MRD) assessment is particularly critical in *BRAF*^V600E^-mutant CRC compared to *BRAF*^V600E^ wild-type cases. Moreover, there remains a need to reconsider follow-up strategies, adjuvant chemotherapy criteria, and administration regimens, as it is unclear whether the current approach for *BRAF*^V600E^ wild-type colorectal cancer is equally applicable to *BRAF*^V600E^-mutant cases. At the same time, advances in genomic analysis have revealed that *BRAF^V600E^*-mutant CRC is not a homogeneous entity. Notably, the BM1 subtype frequently aligns with the CMS4 classification, which is associated with poor prognosis [21,22]. Moreover, recent studies suggest that classifying patients into CD (controlled disease) and UD (uncontrolled disease) groups based on initial treatment response may provide additional prognostic value [23,24]. Moving forward, further exploration of the relationship between disease control patterns (CD vs. UD) and genetic subtypes could lead to refined treatment strategies and more precise prognosis predictions.

### Limitation

This study was conducted as a single-institution observational study with a small sample size, and many cases were treated before BEACON therapy was approved. Although Figure 1 and Figure 2 show statistically significant survival differences, the small sample size warrants cautious interpretation of the log-rank test results, as statistical significance may be overestimated in limited cohort comparisons. To generalize these findings, verification in a larger, multi-institutional setting is necessary.

However, the clear visual differences in survival curves based on cfDNA *BRAF*^V600E^ status suggest that it has significant potential as a clinically relevant biomarker. Given the real-time advantages of cfDNA *BRAF*^V600E^, its integration into clinical practice for treatment monitoring and MRD assessment warrants further investigation.

This study builds on previous research demonstrating the potential of cfDNA *BRAF*^V600E^ as a biomarker in colorectal cancer. By further validating its role in treatment monitoring, postoperative MRD assessment, and conversion surgery selection, these findings contribute valuable insights into the clinical aspects of *BRAF*^V600E^-mutant CRC, which requires a multidisciplinary treatment approach. Future large-scale studies will be crucial to fully establish cfDNA *BRAF*^V600E^ as a standard component of precision medicine in colorectal cancer care.

## 5. Conclusions

This study suggests the potential utility of cfDNA in the treatment of *BRAF*^V600E^-mutant CRC, particularly for treatment monitoring and MRD assessment. As precision medicine in colorectal cancer continues to advance, further developments in diagnostic testing and targeted therapies for *BRAF*^V600E^-mutant CRC are anticipated. With accumulating evidence, improvements in treatment outcomes for *BRAF*^V600E^-mutant CRC are expected, ultimately enhancing patient prognosis and therapeutic strategies.

## Figures and Tables

**Table 1:** Patient characteristics of Cohort 1.

Cohort 1: Stage IV Colorectal Cancer with *BRAF*^V600E^ Mutation		
Age (y/o)	69	(59.0–71.5)
Sex [M/F]	10:4	
Primary site [right/left]	9:5	
Tumor size (mm) ^1^	47.5	(40.3–55)
Histology type [tub/por/muc] ^2^	5:5:4	
Distal metastasis [liver/lung/LN/dissemination/bone] ^3^	6:2:10:1	
Primary tumor resection [yes/no]	10:4	
Surgical procedure (ileocecal resection 3:right hemicolectomy 4:partial resection 3)		
First-line treatment regimen [mFOLFOX6 + Bv/FOLFOXIRI + Bv/mFOLFOX6]	5:6:3	
Pre-treatment CEA (ng/mL)	6:2	(3.5–18.5)
Pre-treatment CA19-9 (U/mL)	87	(2.1–2249.4)
Pre-treatment cfDNA volume (ng/mL)	123	(101.5–145)
	median	(Interquartile range)

^1^ Tumor size in resected cases only. ^2^ Main histological component. ^3^ Multiple sites possible.

**Table 2:** Patient characteristics of Cohort 2.

Cohort 2: *BRAF* ^V600E^ Mutation-Positive Colorectal Cancer with Curative Resection		
Age (y/o)	72	(64.5–82.5)
Sex [M/F]	11:12	
Primary site [right/left]	18:5	
Tumor size (mm)	39	(30–57)
pT [1b/2/3/4a]	2:2:14:5	
pN [0/1a/1b/2a/2b/3]	11:4:3:3:1:1	
Histology type [tub/por/muc] ^1^	12:5:6	
Surgical procedure [ICR/RHC/partial resection/Sigmoidectomy/LAR]	10:8:4:1	
Laparoscopic/open	18:5	
Adjuvant chemotherapy [Yes/No]	9:14	
regimen [CAPOX 5: FOLFOX 1: UFT/UZEL 3]		
Pre-treatment CEA (ng/mL)	4.7	(2.1–8.5)
Pre-treatment CA19-9 (U/mL)	12.7	(7.8–27.7)
Pre-treatment cfDNA volume (ng/mL)	138	(82–151)
	median	(Interquartile range)

^1^ Main histological component.

**Table 3:** Prediction of recurrence using blood samples taken one month after curative resection.

	Relapse	No Relapse		Relapse	No Relapse
*BRAF*^V600E^ positive in cfDNA	4		CEA ≥5.0 ng/mL	2	3
*BRAF*^V600E^ negative in cfDNA	3	16	CEA <5.0 ng/mL	5	13
*p =* 0.004			*p =* 0.58		

## Data Availability

The datasets generated and analyzed in the current study are available from the corresponding author upon reasonable request.
